# Effect of data-collection method on reporting of common mental disorder symptoms and intimate partner violence in Zimbabwe: a cluster-randomized trial

**DOI:** 10.1093/ije/dyaf221

**Published:** 2026-01-14

**Authors:** Victoria Simms, Bridget Kanengoni, Rudo Chingono, Edson T Marambire, Vimbainashe S Mutendereki, Tsitsi Bandason, Celia L Gregson, Claire Calderwood, Leyla Larsson, Rashida A Ferrand, Nicol Redzo, Prosper Chonzi, Katharina Kranzer

**Affiliations:** The Health Research Unit Zimbabwe, Biomedical Research and Training Institute, Harare, Zimbabwe; International Statistics and Epidemiology Group, London School of Hygiene & Tropical Medicine, London, United Kingdom; The Health Research Unit Zimbabwe, Biomedical Research and Training Institute, Harare, Zimbabwe; The Health Research Unit Zimbabwe, Biomedical Research and Training Institute, Harare, Zimbabwe; The Health Research Unit Zimbabwe, Biomedical Research and Training Institute, Harare, Zimbabwe; Musasa Trust, Harare, Zimbabwe; The Health Research Unit Zimbabwe, Biomedical Research and Training Institute, Harare, Zimbabwe; The Health Research Unit Zimbabwe, Biomedical Research and Training Institute, Harare, Zimbabwe; Global Health and Ageing Research Unit, Bristol Medical School, University of Bristol, Bristol, United Kingdom; The Health Research Unit Zimbabwe, Biomedical Research and Training Institute, Harare, Zimbabwe; Clinical Research Department, London School of Hygiene & Tropical Medicine, London, United Kingdom; The Health Research Unit Zimbabwe, Biomedical Research and Training Institute, Harare, Zimbabwe; Division of Infectious Diseases and Tropical Medicine, Ludwig Maximilian University Hospital, Munich, Germany; The Health Research Unit Zimbabwe, Biomedical Research and Training Institute, Harare, Zimbabwe; Clinical Research Department, London School of Hygiene & Tropical Medicine, London, United Kingdom; The Health Research Unit Zimbabwe, Biomedical Research and Training Institute, Harare, Zimbabwe; Harare City Health, Harare, Zimbabwe; The Health Research Unit Zimbabwe, Biomedical Research and Training Institute, Harare, Zimbabwe; Clinical Research Department, London School of Hygiene & Tropical Medicine, London, United Kingdom; Division of Infectious Diseases and Tropical Medicine, Ludwig Maximilian University Hospital, Munich, Germany

**Keywords:** audio computer-assisted self-interview, common mental disorder, health workers, intimate partner violence, Zimbabwe

## Abstract

**Background:**

Screening for sensitive and stigmatized conditions such as mental health or experience of violence is challenging. Audio computer-assisted self-interviewing (ACASI), administered by using a tablet and headphones, may be more sensitive for this purpose than paper-based self-administered questionnaires (SAQ) handed in to project staff. We conducted a methodological cluster-randomized trial in Zimbabwe to compare two methods of screening for common mental disorders (CMD) and intimate partner violence (IPV): ACASI versus SAQ.

**Methods:**

Trial participants were health workers receiving occupational health checks at hospitals and primary health clinics. The unit of randomization was a working day. CMD was measured by using the Shona Symptom Questionnaire, anxiety by using the Generalised Anxiety Disorder-7 questionnaire, and IPV by using the World Health Organization screening questionnaire. The co-primary outcomes were CMD prevalence and the prevalence of any IPV, compared by arm at the cluster level, adjusting for gender and weekend. Secondary outcomes were the prevalence of anxiety and of physical, emotional, and severe physical and sexual IPV.

**Results:**

Between 20 February and 10 June 2022, 1240 participants were enrolled in 71 clusters (workdays), with 77.0% female and 66.4% in clinical-facing roles. The cluster-level geometric mean prevalence of CMD was 19.4% when using ACASI and 14.1% when using SAQ [adjusted risk ratio (aRR) 1.37, 95% confidence interval (CI) 0.99, 1.89; *P *= .056]. ACASI yielded a higher prevalence of overall IPV than the SAQ (cluster-level geometric mean prevalence 40.6% compared with 22.4%, aRR 1.81, 95% CI 1.40, 2.35; *P *< .001), of emotional IPV (aRR 1.66, 95% CI 1.27, 2.17; *P *< .001), and of physical IPV (aRR 1.61, 95% CI 1.16, 2.25; *P *= .005). No differences were seen in the prevalence of severe physical or sexual IPV or anxiety across the trial arms.

**Conclusion:**

Screening for CMD and IPV by using a confidential ACASI method identifies more people who may benefit from care than screening by using SAQ handed in to clinic staff. This may be explained by under-reporting on the SAQ. ACASI is a promising screening method for sensitive issues in healthcare settings.

Key Messages

- We tested the hypothesis that health workers in Zimbabwe would report a higher prevalence of common mental disorders (CMD) symptoms and intimate partner violence (IPV) via audio computer-assisted self-interviewing (ACASI) than via paper-based self-administered questionnaires (SAQ) handed in to a research assistant.
- We found that, in a cluster-randomized trial of ACASI versus SAQ, ACASI was associated with increased prevalence of CMD symptoms and IPV.
- ACASI is recommended for screening for CMD symptoms and IPV because studies that use SAQ may underestimate the prevalence of these conditions and fail to detect people who would benefit from referral.

## Introduction

Screening for common mental disorders (CMD) and intimate partner violence (IPV) can be challenging due to under-reporting, preventing the identification of people who would benefit from support [1]. Audio computer-assisted self-interviewing (ACASI) is a data-collection method in which the participant listens to recorded questions via headphones and then selects a response on a computer screen or tablet. ACASI has been used in many contexts to collect sensitive information, particularly on behaviour counter to social norms such as sexual risk behaviour [2, 3]. Disclosure of sensitive information is increased when the participant has less personal interaction with the data collector [4]. ACASI is thought to remove social pressure and give a sense of privacy [4]. Most of the literature in low- and middle-income countries has found a higher prevalence of sexual risk behaviour (e.g. multiple partners) reported when using ACASI compared with face-to-face interviews [2, 5]. When ACASI has been compared directly with self-administered questionnaires (SAQ), studies in Africa and Asia have found increased reporting of multiple partners, premarital sex, transactional sex, and lower age at sexual debut, but decreased reporting of sexual debut [6–9]. Less evidence is available for the effectiveness of ACASI for other sensitive topics such as experience of abuse [4] or symptoms of mental health disorders, particularly in low-income settings.

Prior to the COVID-19 pandemic, health workers were already at increased risk of both CMD and IPV compared with the rest of the population [10, 11]. The pandemic increased the mental health burden on health workers, leading to a higher prevalence of stress, anxiety, depression, and burnout [12]. COVID-19 also led to reports of an increase in IPV, which itself is a risk factor for depression and anxiety [13, 14]. In the 2015 Zimbabwe Demographic Health Survey, >40% of partnered women reported IPV [15]. During the COVID-19 pandemic in Zimbabwe, a comprehensive health check for frontline health workers provided free, opt-in screening and referral for a range of conditions including screening for IPV and CMD symptoms [16, 17]. Initially, screening for IPV and CMD was conducted by using a self-completed questionnaire that was handed to a research assistant. In the first year, 12.1% of health workers screened positive for CMD [18]. We hypothesized that ACASI would offer greater privacy and a sense of confidentiality, potentially allowing clients to answer sensitive questions more accurately. We conducted a randomized–controlled trial nested within the health-check service, with the aim of assessing whether ACASI increased the screening yield.

## Methods

### Setting and participants

The design and evaluation of the health-check programme have been reported elsewhere [16, 17, 19]. Briefly, the service was provided in a series of tents in the hospital grounds. Clients gave verbal consent and could opt out of any screening test. CMD and IPV screening were performed in the registration tent, with only the participant and a research assistant present. The trial was a methodological cluster-randomized–controlled trial comparing two methods of administration of CMD and IPV screening tools: paper-based SAQ versus ACASI. The unit of randomization (the cluster) was the day of data collection. Days were pre-randomized by V.S. using Stata, stratified by weekday versus weekend (Saturday–Sunday), because participants who worked at weekends might have been systematically different, e.g. including a higher proportion of clinical staff. The health-check service was provided by two teams (Supplementary Table S1). Study staff were given advance schedules indicating each day’s allocation.

Participants were free to attend the health check on any day they chose. Socio-demographic information was collected from all participants by using an interviewer-administered questionnaire before the mental health and IPV screen on a tablet using SurveyCTO (Dobility, Cambridge, MA, USA). Role was classified into clinical (defined as any role involving patient contact) and non-clinical. CMD symptoms were measured by using the 14-item Shona Symptoms Questionnaire (SSQ) on a scale of 0–14, with 0 representing no symptoms and 8 as the cut-off point. A score of ≥11 or a report of hallucinations or suicidal ideation was defined as a ‘red flag’ score. Anxiety symptoms were measured by using the seven-item Generalised Anxiety Disorder questionnaire (GAD-7) on a scale of 0–21, with 0 representing no anxiety symptoms and a cut-off point of 10. The SSQ was developed and validated in Zimbabwe, in Shona and in English translation [20]. The GAD-7 was developed in the USA and validated in Zimbabwe in Shona translation [21].

IPV was measured by using a World Health Organization questionnaire that asked whether the participant had ever experienced each of 13 forms of IPV and whether they had occurred in the previous 12 months [22]. The cut-off point was a response of ‘yes’ to any item. It was administered to all participants who were, or had ever been, married or living with a partner. All staff were trained in the provision of first aid for IPV using the LIVES (Listen, Inquire, Validate, Enhance Safety, Support) approach [23] by a team of experts from the Musasa Trust—a national non-governmental organization providing support to women experiencing IPV.

Data collection followed procedures outlined in the Manual of Operations [19]. Paper versions of the screening questionnaires were prepared in English, Shona, and Ndebele. Translations and back-translations were carried out by research assistants if translated versions did not already exist. Participants read the questionnaire, ticked the answers that corresponded to their experience, then handed it to a research assistant, who immediately calculated the scores, noting those with an SSQ score of ≥8, GAD score of ≥10, IPV, or red flag. The questionnaire was identified by the study ID number, with no name recorded. In the ACASI arm, the screen was self-administered on a pre-programmed tablet using SurveyCTO software with headphones. Questions and possible responses were recorded in English, Shona, and Ndebele by female research assistants. Participants chose their language of preference. When a participant had completed the questionnaire, a final screen appeared that displayed only their ID number and the following information, each with a yes/no response: SSQ score ≥8; GAD score ≥10; IPV; red flag.

Research assistants had ≥2 years’ experience in social science research and an undergraduate degree. They were trained on all study procedures, including how to set up the ACASI tablet and headset, how to instruct the participant and make sure they understood, and what to do when a participant finished. All study staff had open access to standard operating procedures and the Manual of Operations.

Participants who scored ≥8/14 on the SSQ or ≥10/21 on the GAD were offered referral to the counselling services unit (CSU) for counselling. Referral could also be offered for other reasons, such as a positive SARS-CoV-2 test result. Participants who scored ≥11 on the SSQ or who reported a ‘red flag’ symptom were referred to the CSU the same day [18]. Contact details were taken and passed to the CSU. After their last counselling appointment, the counsellor administered the SSQ over the phone if the participants agreed. Participants who reported severe physical or sexual IPV on the screening questionnaire were referred to the Musasa Trust and given a leaflet about its services. Participants were given the hotline number of the Musasa Trust to contact, rather than being called, to maintain confidentiality.

### Data management and analysis

Screening data from the SAQ arm were double-entered into a purpose-designed EpiData form with range and legal checks, and validated. ACASI data were uploaded to the server used for all other study data, cleaned, and checked. Data from the CSU on participant outcomes were collected in Excel and checked. Quantitative analysis was conducted by using Stata v17 at the cluster level due to the number of clusters [24], with a comparison of the trial arms adjusting for gender and weekend by using the ‘clan’ command. The primary outcomes were the cluster-level prevalence of CMD, defined as an SSQ score of ≥8, and the cluster-level prevalence of any violence, defined as answering ‘yes’ to any item on the violence questionnaire. Secondary outcomes were the cluster-level prevalence of each of the four IPV components (emotional, physical, severe physical, and sexual), defined as answering ‘yes’ to any item within the component, and the cluster-level prevalence of anxiety, defined as a GAD-7 score of ≥10. Subgroup analysis of the primary outcomes was conducted by gender a priori because the prevalence of IPV and of CMD symptoms differs by gender. As a sensitivity analysis, all outcomes were analysed at the individual level by using generalized linear log-binomial modelling with standard errors adjusted for clustering by workday. As a post hoc analysis, the proportion of participants who received counselling was calculated and compared at the cluster level. Analysis was carried out by using modified intention to treat; participants were analysed according to the arm that the staff were told to use on that day, even if it was not the one that had been randomly allocated. Sensitivity analysis was carried out by using intention to treat (ITT). Missing data were excluded.

The mean SSQ score using SAQ, prior to the trial, was 3.3 (2.84 SD). With an average cluster size of 20 and an intra-cluster correlation of 0.01, 32 clusters per arm (1280 participants in total) were needed to detect a minimum meaningful difference of 0.5 in the mean SSQ score at 80% power. The trial received ethical approval from the Medical Research Council of Zimbabwe (MRCZ/A/2627) and the London School of Hygiene & Tropical Medicine (22514). Participants gave verbal consent to participate. The requirement for written informed consent was waived for the original study because it was an operational study that was focused primarily on health service provision. Written consent for the trial was waived because it was methodological rather than interventional.

## Results

**Figure 1.**
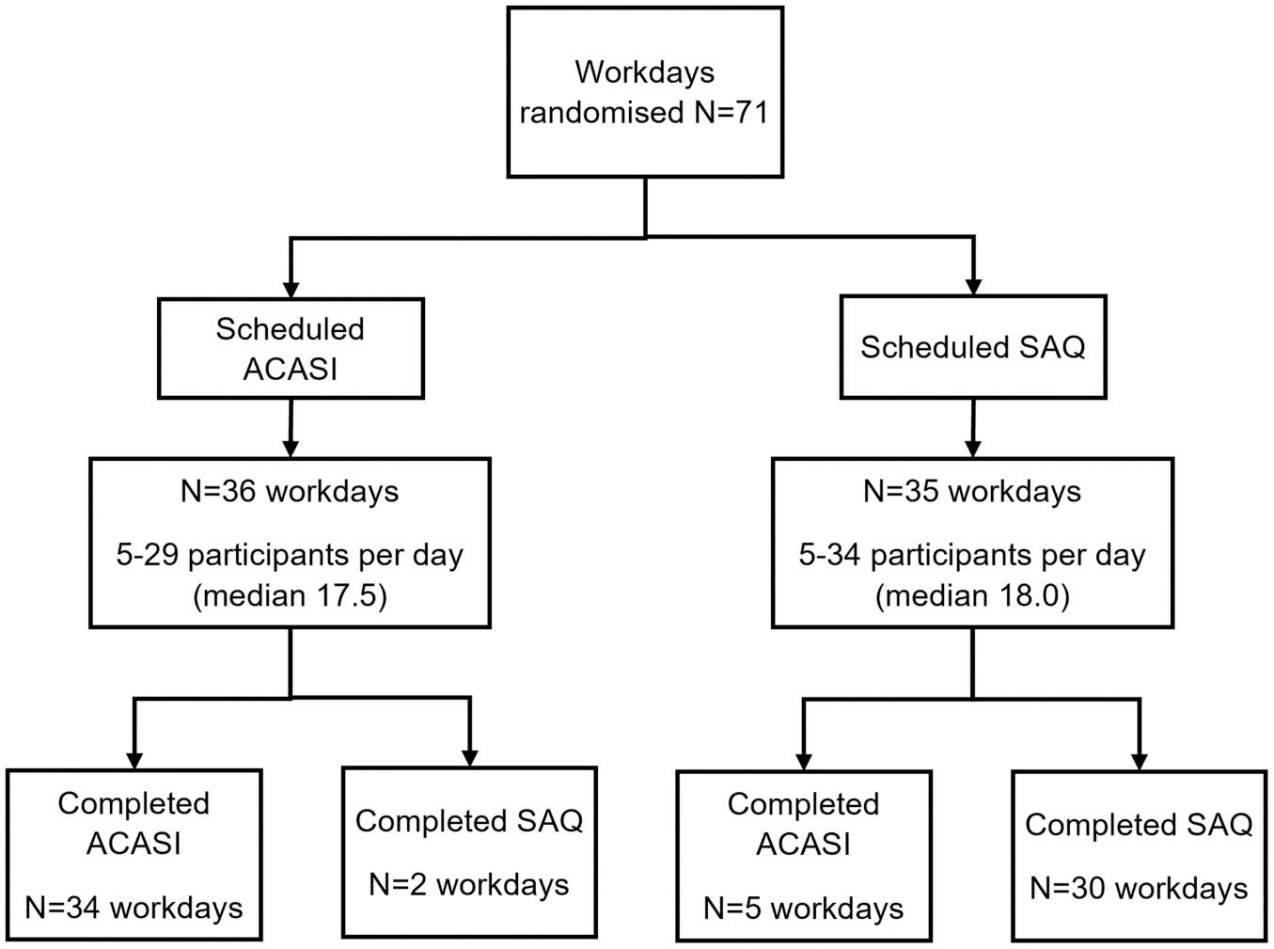
Flowchart of random allocation.

The trial enrolled from 20 February to 10 June 2022, encompassing 71 team workdays (clusters) and 1240 participants (Supplementary Table S1). Seven workdays were incorrectly allocated into the arm that had not been randomly selected (Fig. 1). We analysed these clusters according to the data-collection method that they used, not the one that had been assigned, because the allocation decision was made in advance of meeting any participants and could not have been affected by participant characteristics, which would have introduced bias. Eight participants were allocated to the incorrect arm for that day, although all other participants on that day were dealt with correctly. These were analysed according to the correct arm for the day, following the principle of modified intention to treat.

In the SAQ arm, 567 participants were enrolled during 32 workdays (clusters), with a median of 19 participants (range 3–34) recruited per workday and 2–8 workdays per facility. In the ACASI arm, 673 participants were enrolled during 39 workdays, with a median 17 (range 5–29) recruited per day. IPV records were lost from 2 workdays in the SAQ arm, leaving 30. Two-thirds of participants (66.4%) were in clinical roles (Table 1). In the ACASI arm, 36.6% of the participants were aged ≤30 years, 27.8% were aged 31–40 years, and 35.7% were aged ≥41 years. In the SAQ arm, 31.8% were aged ≤30 years, 31.9% were aged 31–40 years, and 36.3% were aged ≥41 years. In the ACASI arm, 74.6% (*n* = 508/663) of the participants were either married/living with a partner or previously had been (therefore eligible to complete the IPV questionnaire) versus 73.4% (*n* = 359/489) in the SAQ arm. The SSQ and GAD scores by arm are shown in Supplementary Fig. S1.

**Table 1.** Socio-demographic characteristics of participants by trial arm.

		Completed ACASI [*n* (%)]	Completed SAQ [*n* (%)]
		*N* = 673	*N* = 567
Gender	Male	136 (20.2)	149 (26.3)
	Female	537 (79.8)	418 (73.7)
Age (years)	≤30	246 (36.6)	180 (31.8)
	31–40	187 (27.8)	181 (31.9)
	>40	240 (35.7)	206 (36.3)
Healthcare role	Non-clinical	218 (33.2)	186 (34.2)
	Clinical	439 (66.8)	358 (65.8)
	Missing	16	23
Education	Primary	30 (4.5)	17 (3.0)
	Secondary O-level	223 (33.1)	191 (33.7)
	Secondary A-level	87 (12.9)	90 (15.9)
	Diploma	239 (35.5)	208 (36.7)
	University	94 (14.0)	61 (10.8)
Marital status	Married/living with partner	403 (60.8)	294 (60.1)
	Previously married/living with partner but not now	105 (15.8)	65 (13.3)
	Never married/living with partner	155 (23.4)	130 (26.6)
	Missing	10	78
SSQ score	0–7	545 (81.1)	486 (85.7)
	8–14	127 (18.9)	81 (14.3)
	Missing	1	0
GAD score	0–9	614 (92.2)	531 (93.8)
	10–21	52 (7.8)	35 (6.2)
	Missing	7	1
Experienced any IPV^a^	Yes	307 (60.9)	286 (79.7)
	No	197 (39.1)	73 (20.3)
	Missing	4	0
Emotional IPV^a^	Yes	338 (66.9)	294 (81.9)
	No	167 (33.1)	65 (18.1)
	Missing	3	0
Physical IPV^a^	Yes	435 (85.8)	338 (94.2)
	No	72 (14.2)	21 (5.9)
	Missing	1	0
Severe physical IPV^a^	Yes	470 (93.4)	340 (94.7)
	No	33 (6.6)	19 (5.3)
	Missing	5	0
Sexual IPV^a^	Yes	476 (94.6)	345 (96.1)
	No	27 (5.4)	14 (3.9)
	Missing	5	0

a Missing for all participants who did not report that they were or had ever been married/living with partner (165 in ACASI arm, 208 in SAQ arm).

### CMD

The cluster-level geometric mean prevalence of CMD, defined as an SSQ score of ≥8, was 19.4% when ACASI was used and 14.1% when using SAQ (Table 2), and there was weak evidence of an association of CMD with the ACASI trial arm [risk ratio (RR) 1.37, 95% confidence interval (CI) 0.99, 1.89; *P *= .056]. There was no association between the data-collection method and the binary GAD-7 score. ACASI was associated with a half-point increase in the continuous SSQ score (adjusted mean difference 0.54, 95% CI 0.07, 1.02; *P *= .026). There was no association between the trial arm and the continuous GAD score (Table 3). In the gender-stratified analysis (Table 4), ACASI was associated with an increased prevalence of CMD in women but not in men, although there was no evidence of interaction by gender (*t* = –0.51, *P *= .62).

**Table 2.** Prevalence and cluster-level prevalence of primary and secondary binary outcomes by trial arm and results of logistic regression adjusted for gender.

	Overall prevalence (%)		Cluster-level geometric mean prevalence (%)		*N* clusters	Risk ratio (95% CI)	*P* value
	ACASI	SAQ	ACASI	SAQ			
SSQ ≥ 8	18.9	14.3	19.4	14.1	71	1.37 (0.99, 1.89)	.056
GAD ≥ 10	7.8	6.2	9.2	8.4	71	1.08 (0.77, 1.51)	.66
All violence	39.1	20.3	41.1	20.8	69	1.99 (1.55, 2.57)	<.001
Emotional violence	33.1	18.1	34.8	18.9	69	1.85 (1.41, 2.43)	<.001
Physical violence	14.2	5.9	15.9	8.8	69	1.79 (1.30, 2.47)	<.001
Severe physical violence	6.6	5.3	9.2	9.2	69	1.05 (0.75, 1.46)	.79
Sexual violence	5.4	3.9	8.3	6.9	69	1.32 (0.86, 2.01)	.19

**Table 3.** Cluster-level mean of continuous trial outcomes by trial arm and mean difference between arms adjusted for gender.

	Cluster-level mean		Adjusted mean difference (95% CI)	*P* value
	ACASI	SAQ		
SSQ score (range 0–14)	4.49	3.93	0.54 (0.07, 1.02)	.026
GAD score (range 0–21)	3.50	3.09	0.39 (–0.10, 0.89)	.12

**Table 4.** Prevalence and cluster-level geometric mean prevalence of SSQ score of ≥8 and of IPV by trial arm, stratified by gender, with results of logistic regression stratified by gender.

Outcome	Gender	Overall prevalence		*N* clusters	Cluster-level geometric mean prevalence		RR (95% CI)	*P* value
		SAQ	ACASI		SAQ	ACASI		
SSQ ≥ 8	Men	15.4	14.0	69	24.2	27.7	1.13 (0.79, 1.61)	.51
	Women	13.9	20.2	71	15.7	21.4	1.39 (1.01, 1.91)	.042
All violence	Men	18.6	44.7	59	28.7	67.1	2.40 (1.72, 3.37)	<.001
	Women	20.9	37.8	69	23.4	41.0	1.75 (1.33, 2.29)	<.001

**Figure 2.**
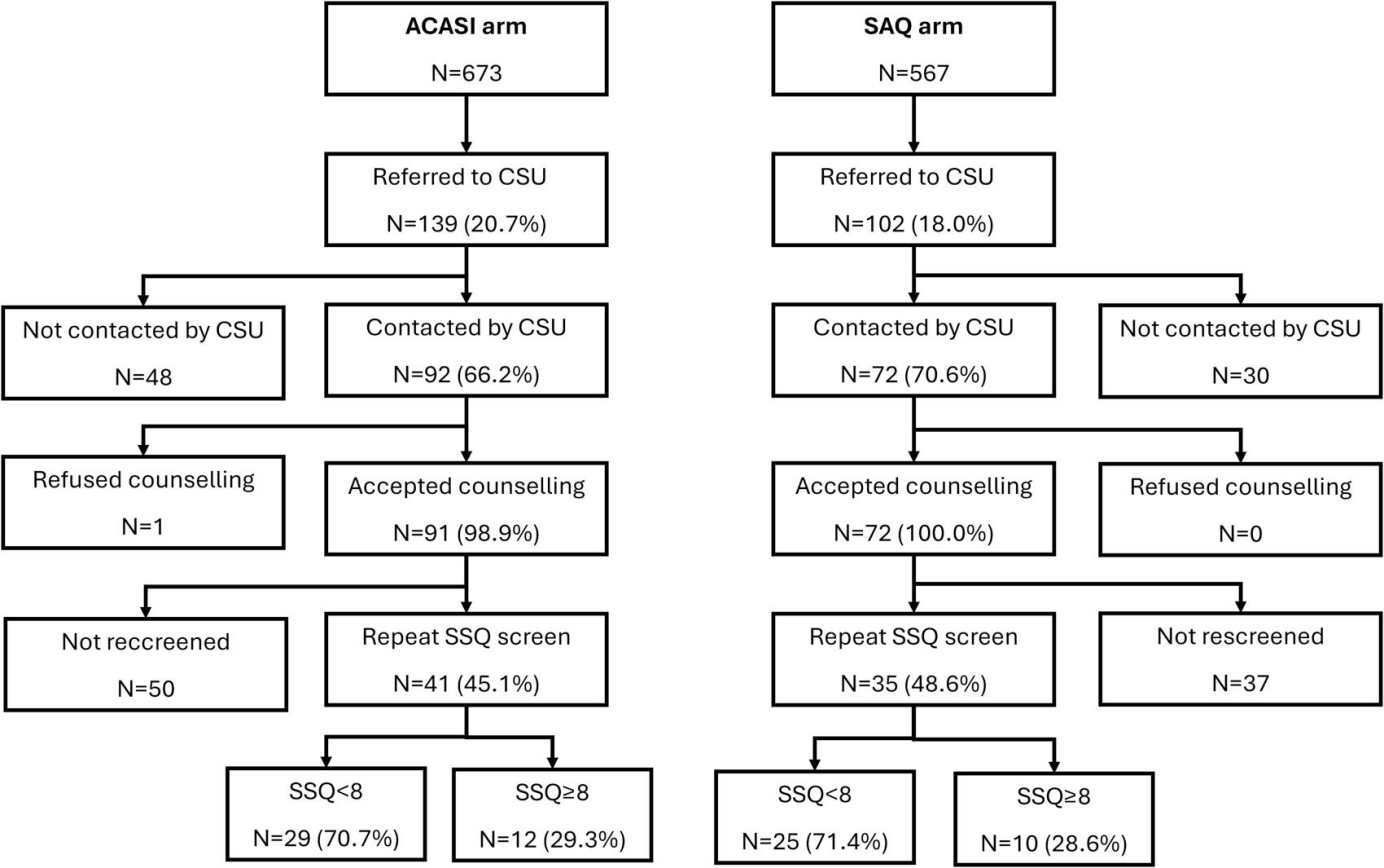
Flowchart of referral to the CSU, uptake of counselling, and results of repeat SSQ screening by trial arm.

In the ACASI arm, 139 participants (20.7%) were referred to the CSU for counselling, including 121/127 participants who had an SSQ score of ≥8 (Supplementary Table S2). The CSU successfully contacted 92/139 (66.2%) participants, of whom 91 accepted counselling (65 had one session, 22 had two sessions, and 2 had three sessions). After counselling, 41/91 (45.1%) had a repeat SSQ screen administered, of whom 12 (29.3%) still had an SSQ score of ≥8 (Fig. 2). In the SAQ arm, 102 participants (18.0%) were referred to the CSU, including78/81 of those with an SSQ score of ≥8. Of 102 referred participants 72 (70.6%) were contacted by the CSU, all 72 accepted counselling (53 had one session, 18 had two sessions, and 1 had three sessions), and 35 were rescreened with the SSQ, of whom 10 (28.6%) had an SSQ score of ≥8. There was no evidence of a difference between arms in the proportion of all participants who received counselling [cluster-level geometric mean prevalence 13.2% vs 12.1%, adjusted risk ratio (aRR) 1.09, 95% CI 0.70–1.71; *P *= .71].

### IPV

ACASI data collection was associated with increased reporting of all forms of IPV (cluster-level geometric mean prevalence 41.1% compared with 20.8%, RR 1.99, 95% CI 1.55, 2.57; *P *< .001) (Table 2). Emotional IPV was the most common form of IPV reported, followed by physical, severe physical, and sexual IPV. Among women using ACASI, 26.9% of the respondents reported emotional violence, 11.4% physical, 6.4% severe physical, and 5.4% sexual violence. In men using ACASI, 29.4% reported emotional violence, 8.4% physical, 4.5% severe physical, and 2.2% sexual IPV. The cluster-level geometric mean prevalence of emotional IPV was 34.8% collected by using ACASI compared with 18.9% collected by using SAQ (RR 1.85, 95% CI 1.41, 2.43; *P *< .001). The prevalence of physical IPV was also associated with the trial arm, at 15.9% when using ACASI compared with 8.8% (RR 1.79, 95% CI 1.30, 2.47; *P *< .001). There was no association between screening method and report of severe physical or sexual IPV.

ACASI was associated with increased prevalence of reports of all forms of IPV in both men and women (Table 4) with no evidence of interaction by gender (*t* = 0.15, *P *= .88). In men, the geometric mean prevalences of both outcomes were very different from the overall prevalences by arm because the number of men per workday was often small (mean cluster size 4.4) and many clusters had no cases. Only 59 clusters had any men who completed the IPV questions and in only 38 did any men report IPV.

Sensitivity analysis at the individual level found comparable results to that at the cluster-level analysis (Supplementary Table S3). Sensitivity analysis using ITT, in which the seven clusters that used the wrong method were analysed in the arm that had been randomly selected for them, found no evidence of an association between CMD symptoms and the trial arm (Supplementary Table S4). The results for IPV and anxiety remained similar to those in the primary analysis.

## Discussion

Use of ACASI was associated with increased reporting of CMD and IPV. As a result, more participants who used ACASI were offered the opportunity to take up CMD counselling or referral to an IPV service, which they would not have received had they been screened by using questionnaires. However, the proportions of participants who actually received counselling were no different between the arms.

The IPV prevalence in men in the ACASI arm was high. There is emerging evidence, mainly from the USA, that the prevalence of IPV in men is higher than previously thought [25–27]. Men may find it particularly difficult to report IPV. Being perceived as a victim of violence goes against traditional masculine norms of toughness and social status [28].

These findings agree with those in the literature in a broad range of settings and populations that IPV is reported more frequently when using ACASI than when using other methods. A systematic review of IPV screening methods identified two randomized controlled trials (RCTs), both in North America, that compared a computer-assisted self-administered (CASI) method with a self-administered paper-based screen method [29, 30]. Reported IPV prevalence was higher when using the CASI method, although the evidence was weak (pooled odds ratio (OR) 1.23, 95% CI 0.92, 1.64) [1]. Sexual violence was more frequently reported in ACASI than in face-to-face interviews by primary school students in Uganda [31] and by sex workers in Kenya [32]. By contrast, a study in India found that domestic violence was less frequently reported when using ACASI compared with face-to-face interviews [33]. The authors suggested that unfamiliarity with the technology (only 7% had ever used a computer) may have hampered the use of ACASI. Two systematic reviews of ACASI have also found that sexual behaviours and experiences (such as ‘ever had sex’, ‘multiple partners’, and ‘forced sex’) were more frequently reported when using ACASI than when using other methods, including face-to-face interviews [2, 5], although a third systematic review found mixed evidence [3].

There is comparatively little research into the effect of ACASI on CMD symptom reporting, but the available evidence supports our finding that symptoms are reported more frequently with ACASI. A study in the USA found that ACASI identified a higher prevalence of mental disorder symptoms than did paper questionnaires [34]. In Zimbabwe, ACASI has been used to collect sensitive information since at least 1998 [35–37]. A study in Zimbabwe compared four CMD screening methods among adolescents and ACASI recorded the highest symptom prevalence [6].

ACASI has other potential advantages apart from its higher yield, including increased acceptability [38] and the freeing-up of provider time by transferring the screening task from the provider to the participant. In the context of this study, no time-saving benefit was expected because the providers remained present to explain the process and provide assistance.

Screening is only effective if it leads to an appropriate response [39]. In this study, there was no evidence of a difference between arms in uptake of counselling or IPV referral or in mental health outcomes. Participants who accepted a referral to the CSU were asked for multiple phone numbers, including the number of a trusted friend. Nonetheless, despite repeated attempts, the CSU staff were only able to contact 68.0% (164/241) of the participants who were referred for counselling because of their SSQ or GAD score, although they contacted 94.1% (16/17) of the participants referred for other reasons. A possible explanation is that participants who did not feel they needed counselling did not respond to attempts to contact them—a form of soft refusal. In the ACASI arm, a higher proportion of referrals to the CSU were based on the SSQ or GAD score rather than for other reasons and the uptake of counselling was lower than that in the SAQ arm (64.4% vs 68.5%). The reason for which the trial found no difference between the arms in the referral uptake may have been that some of the participants who screened positive did not feel that they would benefit from counselling. Similarly, women referred due to IPV may not have felt able to take up the referral at that point [39]. Only 76 participants were rescreened using the SSQ after counselling, comprising 46.6% of all participants given counselling and 32% of those referred for counselling. Mental health at follow-up was not planned as a trial outcome and the trial was not powered to detect an effect.

There are logistical implications to using ACASI in low-income countries [35, 40]. Users may be unfamiliar with electronic devices such as tablets, yet are reluctant to ask for help. The study participants, who were all employed, probably had higher levels of education and digital literacy than the average Zimbabwean population. However, all staff were eligible, including those in lower-paid work such as porters and cleaners, and 37.2% of the participants had an O-level education or less. Digital literacy is likely to have varied widely. Service staff reported that many participants required their assistance to use the tablet, although this information was not systematically captured. The assistance may have influenced participant responses, as in the 2011 study in India [33]. As smartphone and tablet ownership increases in low-income settings, the technology is likely to become more familiar.

The observed increase in the prevalence of CMD and IPV when using ACASI could have been due to an increase in the false-positive rate rather than a decrease in the false-negative rate. However, it is more probable that the increased anonymity of ACASI would have enabled participants to report their experience more fully. In this case, the tools were used as screening instruments to inform referral for counselling, so increased sensitivity would have been desirable even at the expense of decreased specificity.

The study’s strengths are that it was a multisite, controlled trial that made use of the implementation design to embed cluster randomization into service delivery. Staff were experienced social science researchers who were trained to support the participants and not interfere with their responses. The study has some limitations. Seven clusters were incorrectly assigned to the wrong trial arm, although this is unlikely to have caused bias because the allocation of the data-collection method was made before any information was known about that day’s participants. Some clusters were small, particularly in the gender-specific analysis, in which 20 clusters had no men who reported IPV, causing the cluster-level geometric prevalence to differ from the crude prevalence. Those attending the service may not have been representative of all health workers at the facilities. However, even if this were the case, it should not have affected the results of the trial, which calculated relative risks. Other limitations are that post-counselling outcomes were only recorded for 47% of the participants who accepted counselling and those who did not accept counselling were not rescreened. Health workers might have based their decision of when to attend the health check upon whether they had heard from colleagues that ACASI was being used that day, but this is unlikely, as no difference in uptake between the arms was observed. The trial protocol received ethical approval from the MRCZ but was not preregistered with a clinical trials registry.

The feasibility and acceptability of the two methods were not assessed and we did not collect data on the time taken to administer the screening. A costing study was not done and the scalability is not known. The additional costs of ACASI consist of the cost of tablets, headsets, electricity and charging time for the tablets, programming time, and software. These are set against the costs of printing, paper, and data-entry time for the SAQ. ACASI is likely to be more cost-effective at scale because certain costs such as the programming time will not scale up. In conclusion, the administration of screening for CMD and IPV when using ACASI elicited more potential CMD cases than did screening with questionnaires, but did not increase the number of participants who received counselling.

## Ethics approval

The trial received ethical approval from the Medical Research Council of Zimbabwe (MRCZ/A/2627) and the London School of Hygiene & Tropical Medicine (22514).

## Supplementary Material

dyaf221_Supplementary_Data

## Data Availability

Data are not available for sharing because the participants gave verbal, not written, consent to participate.
