# Supplementary material for: Effect of data-collection method on reporting of common mental disorder symptoms and intimate partner violence in Zimbabwe: a cluster-randomized trial
Source: Int J Epidemiol. 2026 Jan 14;55(1):dyaf221. doi: 10.1093/ije/dyaf221 (PMC12802919; doi:10.1093/ije/dyaf221)
Supplement: dyaf221_Supplementary_Data [file dyaf221_supplementary_data.docx]

**Supplementary material**

Table S1: Data collection by team, location and time

| Location | Team | Dates | Number of days | Number of participants enrolled |
| --- | --- | --- | --- | --- |
| Wilkins ID Hospital | A | 22 Feb-3 Mar | 8 | 114 |
| Mvurwi Hospital | A | 7 Mar-11 Mar | 5 | 39 |
| Tariro Polyclinic | A | 14 Mar-17 Mar | 4 | 35 |
| Thorngrove ID Hospital | B | 20-22 Feb  25-26 Feb | 5 | 49 |
| Ekusileni Hospital | B | 23 Feb-24 Feb | 2 | 41 |
| St Luke’s Hospital | B | 6-13 Mar | 8 | 157 |
| Gwanda PH | B | 22 Mar-29 Mar | 8 | 176 |
| Masvingo PH | B | 7-14 April | 8 | 163 |
| Mutare PH | B | 24 April-1 May | 8 | 161 |
| Gweru PH | B | 17-24 May | 8 | 167 |
| Chinhoyi PH | B | 3-10 June | 7 | 138 |
| Total |  |  | 71 | 1240 |

*Team A was based in Harare and worked Mondays to Fridays in locations within a day’s commuting distance from Harare. Team B was national and provided the health check-up in provincial hospitals. Team B worked a 10-day schedule (8 days of the health check-up plus 1 day for travel each way).*

*ID: Infectious Disease. PH: Provincial Hospital*

Table S2: criteria for referral and counselling uptake by trial arm

| Criteria for referral | SAQ arm | | ACASI arm | |
| --- | --- | --- | --- | --- |
|  | Referred | Uptake of counselling | Referred | Uptake of counselling |
| SSQ≥8 and GAD≥10 | 20 | 12 (60%) | 39 | 25 (64%) |
| SSQ≥8 only | 58 | 40 (69%) | 82 | 52 (63%) |
| GAD≥10 only | 14 | 11 (79%) | 11 | 8 (73%) |
| Other | 10 | 9 (90%) | 7 | 7 (100%) |

ACASI: audio computer-assisted self-interview. GAD: Generalised Anxiety Disorder questionnaire. IPV: intimate partner violence. SAQ: self-administered questionnaire. SSQ: Shona Symptom Questionnaire.

Table S3: individual level analysis of outcomes using generalised linear log-binomial modelling adjusting for clustering and sex

|  | N clusters | RR (95% CI) | P-value | Rho |
| --- | --- | --- | --- | --- |
| SSQ≥8 | 71 | 1.31 (0.95, 1.82) | 0.10 | 0.06 |
| GAD≥10 | 71 | 1.2 (0.87, 1.79 | 0.23 | 0 |
| All violence | 69 | 1.94 (1.49, 2.52) | <0.001 | 0.02 |
| Emotional violence | 69 | 1.85 (1.40, 2.44) | <0.001 | 0.01 |
| Physical violence | 69 | 2.40 (1.51, 3.81) | <0.001 | 0.03 |
| Severe physical violence | 69 | 1.22 (0.72, 2.07) | 0.46 | 0 |
| Sexual violence | 69 | 1.33 (0.56, 3.16) | 0.52 | 0.13 |

ACASI: audio computer-assisted self-interview. GAD: Generalised Anxiety Disorder questionnaire. RR: risk ratio. SAQ: self-administered questionnaire. SSQ: Shona Symptom Questionnaire.

Table S4: sensitivity analysis of binary outcomes using ITT

|  | Overall prevalence, % | | Cluster-level geometric mean prevalence, % | | N clusters | Risk ratio (95% CI) | P-value |
| --- | --- | --- | --- | --- | --- | --- | --- |
|  | ACASI | SAQ | ACASI | SAQ |  |  |  |
| SSQ≥8 | 18.8 | 14.7 | 19.1 | 14.7 | 71 | 1.30 (094, 1.79) | 0.12 |
| GAD≥10 | 6.9 | 7.2 | 8.4 | 9.4 | 71 | 0.87 (0.62, 1.22) | 0.42 |
| All violence | 39.6 | 22.1 | 41.6 | 22.6 | 69 | 1.85 (1.42, 2.40) | <0.001 |
| Emotional violence | 33.6 | 19.5 | 35.0 | 20.5 | 69 | 1.72 (1.30, 2.27) | <0.001 |
| Physical violence | 13.6 | 7.5 | 14.7 | 10.3 | 69 | 1.42 (1.09, 1.99) | 0.045 |
| Severe physical violence | 6.2 | 5.9 | 8.4 | 9.7 | 69 | 0.86 (0.62, 2.20) | 0.36 |
| Sexual violence | 5.5 | 3.9 | 8.0 | 7.3 | 69 | 1.06 (0.70, 1.62) | 0.78 |

ACASI: audio computer-assisted self-interview. GAD: Generalised Anxiety Disorder questionnaire. RR: risk ratio. SAQ: self-administered questionnaire. SSQ: Shona Symptom Questionnaire.


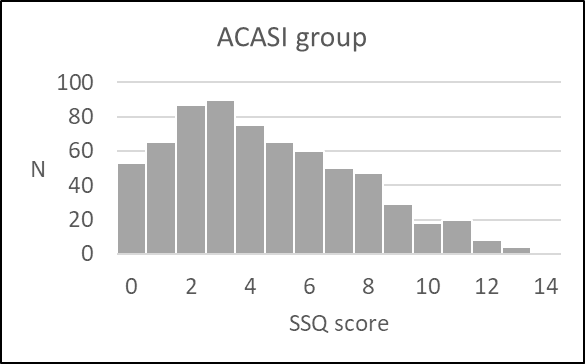

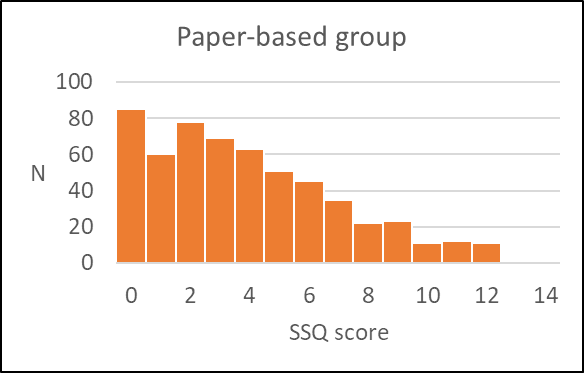

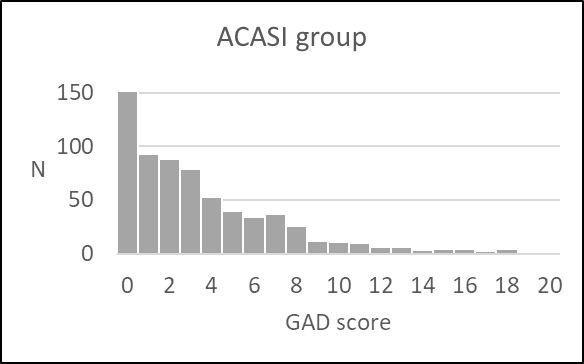

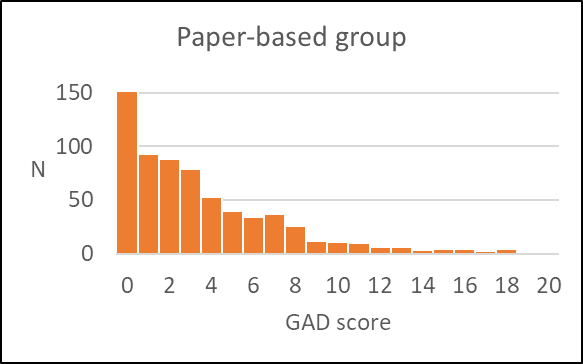


Figure S1: Distribution of SSQ and GAD_7 scores by trial arm

ACASI: audio computer-assisted self-interview. GAD: Generalised Anxiety Disorder questionnaire. RR: risk ratio. SAQ: self-administered questionnaire. SSQ: Shona Symptom Questionnaire.
